# Utilization and compliance with iron supplementation and predictors among pregnant women in Southeast Ethiopia

**DOI:** 10.1038/s41598-022-20614-9

**Published:** 2022-09-28

**Authors:** Girma Beressa, Bikila Lencha, Tafese Bosha, Gudina Egata

**Affiliations:** 1 School of Health Sciences, Madda Walabu University, P.O. Box 302, Goba, Ethiopia; 2 School of Nutrition, Food Science and Technology, Hawassa University, P.O. Box 05, Hawassa, Ethiopia; 3 School of Public Health, Addis Ababa University, P.O. Box 1176, Addis Ababa, Ethiopia

**Keywords:** Biochemistry, Health care, Medical research

## Abstract

Anemia is predicted to affect 38% (32 million) of pregnant women worldwide. However, evidence for utilization and compliance with iron supplementation and predictors during pregnancy in low-income countries, including Ethiopia, is sparse and inconclusive. Therefore, we aimed to assess utilization and compliance with iron supplementation and predictors among pregnant women in Robe Town, Southeast Ethiopia. A community-based cross-sectional study was employed among randomly selected 445 pregnant women attending antenatal care at health facilities from May to July 2015. A systematic random sampling was used to select respondents. Data were collected using a pre-tested, interviewer-administered, structured questionnaire. Bivariable and multivariable logistic regression analyses were conducted to identify predictors of compliance with iron supplementation. An odds ratio, along with a 95% confidence interval (CI), was used to estimate the strength of the association. In this study, 54% [95% CI (49.4, 58.4%)], 45.2% [95% CI (40.9, 49.4%)], 4.3% [95% CI (2.5, 6.3%)], and 2.2% [95% CI (1.1, 3.6%)] of women received iron supplements during their first, second, third, and fourth antenatal care visits, respectively. The level of compliance with iron supplementation was 92.4% [95% CI (89.9, 94.6%)]. Having a formal education (AOR = 4.45, 95% CI 1.41, 13.99), being in the high wealth quintile (AOR = 0.18, 95% CI 0.05, 0.68), medium wealth quintile [(AOR = 0.33, 95% CI (0.11, 0.98)], receiving iron supplements for free (AOR = 3.77, 95% CI 1.33, 10.69), not experiencing discomfort related to iron supplements intake (AOR = 2.94, 95% CI 1.17, 7.39), having comprehensive knowledge about anemia (AOR = 2.62, 95% CI 1.02, 6.70), being knowledgeable about iron supplements (AOR = 3.30, 95% CI 1.12, 9.76), having information about importance of iron supplementation during pregnancy (AOR = 2.86; 95% CI 1.04, 7.87), and ever being visited by urban health extension workers (AOR = 0.31; 95% CI 0.12, 0.83) was significantly associated with compliance with iron supplementation during pregnancy. The utilization of iron supplementation during pregnancy was low, with relatively high compliance with the supplements. Thus, comprehensive nutrition education and free provision of iron supplementation are crucial tools to increase utilization and compliance with iron supplementation during pregnancy. Further research with a strong study design using golden standard methods is warranted.

## Introduction

Iron-deficiency anemia is one of the most common nutritional disorders worldwide, affecting populations in both high-income countries (HICs) and low and middle-income countries (LMICs)**.** Anemia is predicted to affect 38% (32 million) of pregnant women worldwide by 2025^1^. However, the prevalence of anemia is increasing in LMICs with a rate of 35.6–76.7%^2–5^. Africa has the highest prevalence of anemia, with 57% of pregnant women^6^. Several studies carried out in Ethiopia also revealed that the prevalence of anemia among pregnant women ranges from 21 to 54%^7–11^. Maternal anemia is associated with mortality and morbidity for the mother and baby, including the risk of miscarriages, stillbirths, prematurity, and low birth weight. It impairs children’s development and learning too, further impacting economic productivity and development^12^.

By 2025, the World Health Organization (WHO) aims to reduce anemia in women of reproductive age by half^12^. In Ethiopia, nutrition is integrated into the health sector transformation plan in the form of micronutrient interventions to prevent the occurrence of anemia and improve the nutritional condition of mothers during pregnancy^13^. Maternal and child mortality remain high in Ethiopia, with 412 maternal deaths per 100,000 live births and 67 child deaths per 103 live births reported^14^. Taking this into account, the Ethiopian government has demonstrated its commitment to averting nutritional problems by adopting food and nutrition policies, strategies, and programs. In addition, the government designed the Seqota declaration to eliminate stunting by 2030^15^. Various factors, such as socio-demographic and health factors, determine the compliance with iron supplementation among pregnant women^16^. All pregnant women in places where anemia is common should take iron supplements, according to the WHO^17^. Despite the WHO's recommendation, iron supplementation is nevertheless uncommon in many countries, particularly in those with limited resources^18^.

The national guideline for the control and prevention of micronutrient deficiencies emphasized the necessity of daily iron supplementation for at least 6 months during pregnancy and 3 months after delivery, in line with WHO recommendations^19^. By 2015, the national nutrition strategy (NNS) aims to increase to 50% the proportion of women who receive iron supplements for more than 90 days during pregnancy and the postpartum period^20^.

In the previous 5 years, only 17.3% of women used iron supplements during their most recent pregnancy, and only 0.4% supplemented for 90 days or more. Even though iron supplementation is thought to be an important aspect of prenatal care (ANC), only 37% of women who had ANC received it^14^. Low adherence has an impact on the level of energy and productivity, cognitive and physical development, and immune function^21^. Several studies reported that the compliance rate of iron supplements during pregnancy was low. For example, the compliance rates vary from 12 to 93%^22–26^.

Previous studies have employed a cut-off point of ≥ 90 days to determine adherence to iron supplementation during pregnancy in sub-Saharan Africa (SSA)^20,27–30^. However, many women in SSA countries do not take iron supplements because of socio-demographic and economic factors^22,27,31–33^, lack of knowledge about anemia, inadequate supply of iron tablets, poor utilization of ANC services, inability to pay for supplementation, misinformation about the benefit of supplementation, complaints about side effects, forgetfulness, and poor counseling^20,27,34,35^.

Although prenatal iron supplementation was an integral part of antenatal care and was provided free of charge in Ethiopia, the existing compliance rate of iron supplements varies from 28.1 to 87.6%^36,37^. Nevertheless, evidence on utilization and compliance with iron supplementation and predictors among pregnant women in low-income countries like Ethiopia, in general, and in the study context, in particular, is sparse and inconclusive. The findings of this study could be used by policymakers to improve utilization and compliance with iron supplementation during pregnancy. Therefore, we aimed to assess the utilization and compliance with iron supplementation and predictors among pregnant women in Robe town, Bale zone, Southeast Ethiopia.

## Methods

### Study design, area, and participants

A community-based cross-sectional study design was used among pregnant women attending antenatal care at health facilities in Robe Town, Bale Zone, Southeast Ethiopia from May to July 2015. The majority of the inhabitants were Muslim (48%), Orthodox (45%), and Protestant (6%). There was one government district hospital. Besides, there were 15 private clinics, 12 drug vendors, and one health center. Five of the 12 drug vendors served rural areas. All health facilities provide antenatal care (ANC) visits. There were also primary and secondary schools, a teachers' training college, and Madda Walabu University in the town. The Robe was entirely engulfed by peasant associations. The child-bearing age women counted were 13,685. The estimated number of pregnant women was 2146^38,39^.

All pregnant women who attended the ANC clinic were the source population. All randomly selected pregnant women attending the ANC clinic who fulfilled the inclusion criteria and who received the iron supplement for at least 1 month before the date of interview were included in the study. Pregnant women who did not respond to the interview questions due to severe psychiatric illness, were unable to speak or hear, were excluded from the study.

### Sample size determination and sampling techniques

The sample size was calculated using the G-power software version 3.1, assuming an effect size of 0.25, a 95% confidence interval, a precision of 0.05, and a power (1 − β) of 80%^40^. The computed sample size was 206. Using a design effect of 2 and adding a 10% non-response rate, the final sample size was *454*.

The three kebeles (the smallest administrative unit in Ethiopia) were selected at random and their respective numbers of pregnant women were obtained from the study town health office. Then, the sample size was proportionally allocated to each kebele, and the pregnant women were selected using a systematic random sampling technique. In the event that two pregnant women were present in the house, we used the lottery method to select the pregnant women. If a woman was absent from her house during the interview, an eligible pregnant woman in the next house in serial number was interviewed. The house of an absent pregnant woman was revisited the next day.

### Data collection

An interviewer-administered, structured questionnaire was used to collect data. The questionnaire was comprised of questions related to socio-demographic and economic factors such as age, marital status, religion, ethnic group, head of household, occupation of the women and husband, educational status of women and husband, wealth index, and family size.

Iron supplements and health-related factors included the experience of discomfort related to iron supplement intake encountered, a dose of iron supplement, knowledge of anemia's causes, symptoms, and prevention, knowledge of iron supplements, beliefs that iron supplement intake may harm the fetus, ever forgotten iron supplement intake, knowledge of anemia, information about iron supplementation, and source of information. Questions related to health care delivery system-related factors were: a source of iron supplement distance from the nearest health facility urban health extension worker visit and ANC services. Obstetric-related factors were also birth order, intended pregnancy, gestational age, the experience of serious pregnancy-related complications, and parity.

To ensure the quality of the data, the questionnaire was initially developed in English and then translated into the local language "*Afan Oromo*" and back-translated into English by independent language experts to ensure its consistency. The questionnaire was also pretested on 5% of the total estimated sample size, having similar characteristics to the study population in a different adjacent setting. The authors and statistician reviewed the completed questionnaire to ensure its validity in terms of content. Finally, we modified the items in light of the findings of the pre-test and expert reviews. The training was given to data collectors, level IV nurses, and supervisors on the objectives of the study, data collection instruments, and principles of research ethics to minimize interviewers' bias. Data supervisors closely supervised the data collectors daily for the successful completion of the questionnaire and timely action otherwise. The study participants were interviewed at their homes to improve the response rate.

### Operational definitions

Utilization of iron supplementation and compliance with iron supplements during pregnancy were outcome variables. In this study, the *utilization* of iron supplementation during pregnancy was determined by the proportion of pregnant women who had been supplemented previously for at least 1 month before the date of the interview. It was measured by how many pregnant women used iron supplements. *Compliance with/adherence* to iron supplements during pregnancy was understood in such a way that a pregnant woman was said to be compliant with/adherent to iron supplements if she took the supplement for at least 4 days a week during pregnancy in the previous month preceding the survey; otherwise, they were classified as non-compliant/adherent to iron supplements. Self-report compliance/adherence was estimated by asking a woman how many times she took a supplement per week in the previous time^6,41,42^.

*Comprehensive knowledge of anemia during pregnancy* was measured in this study in which respondents who were aware of anemia and knew at least one of its major causes, symptoms, and consequences during pregnancy were said to be knowledgeable about anemia. On the contrary, knowledge of iron supplementation was measured based on a set of four questions including (1) awareness of iron supplementation in pregnancy, (2) reasons for iron supplementation, (3) possible side effects of oral iron preparation on women, and (4) possible effects of iron deficiency. Respondents who correctly answered 3 or 4 of the above questions were considered to have good knowledge of anemia during pregnancy, whereas those who answered two or fewer correctly were said to have poor knowledge^24^.

*The knowledge of iron supplementation during pregnancy* was based on the participants' responses to a set of four questions, including (1) awareness of iron supplementation in pregnancy, (2) reasons for iron supplementation, (3) possible side effects of oral iron preparation on the women, and (4) possible effects of iron deficiency on pregnancy. Respondents who answered correctly to 3 or 4 of the above questions were considered to have good knowledge of iron supplementation in pregnancy, whereas those who answered correctly to two or fewer were said to have poor knowledge^24^.

The economic status of respondents was ranked and ordered into tertiles as rich, medium, and poor using the wealth index. The wealth index was assessed by computing principal component analysis using household asset items and other variables^14^.

### Statistical analysis

Data were checked for completeness, consistency, and accuracy. Then, the data were entered, cleaned, and analyzed using SPSS version 20 computer software. Descriptive statistics such as frequencies, percentages, and means were computed for selected explanatory variables. The normality of the data distribution was assessed using the Kolmogorov–Smirnov test. A bivariable logistic regression analysis was conducted to see the association between each predictor and compliance with iron supplements (Yes = 1, No = 0). Variables with a *p*-value of ≤ 0.25 were included in multivariable logistic regression analyses to control for all possible confounder effects. Multicollinearity was tested among all independent variables using a correlation matrix (R). The highest correlation between the independent variables was 0.16, which was below the cut-off points. The crude odds ratio (COR) and adjusted odds ratio (AOR) were conducted to estimate the strength of the association between the predictors and the outcome variable. The goodness of fit of the final logistic model was tested using the Hosmer and Lemeshow's test at a *p*-value of greater than 0.05. The statistical significance of the association was declared at a *p*-value of less than 0.05.

### Ethical considerations

The current study was ethically approved by the Institutional Review Board of Hawassa University before the start of the study (Protocol Number: IRB/07/07). A permission letter was obtained from the Bale Zone and Robe district health offices. All methods were performed in accordance with the relevant tenets of the Helsinki Declaration^43^. Informed written consent was obtained from each respondent. The privacy of the respondents was respected. Confidentiality was maintained throughout the entire process of data collection and management.

## Results

### Socio-demographic and economic characteristics

A total of 445 out of 454 pregnant women, were included in the study, yielding a response rate of 98%. Nearly half 214 (48%) of pregnant women were less than or equal to 24 years of age, with a mean (± SD) age of 25 (± 5.42) years. Almost all (98%) of the pregnant women were married, and half of them were Muslims. The majority of the respondents (82.5%) were from the Oromo ethnic group. Half of the households were headed by husbands. More than half (58.4%) of the respondents were housewives, whereas (2.7%) were farmers. The majority of the respondents (94.6%) were literate, and about (30.8%) of the households had high wealth quintiles (Table 1).

**Table 1:** Socio-economic and demographic characteristics of pregnant women attending antenatal care at Robe Town, Bale zone, Southeast Ethiopia, 2015 (n = 445).

Variables	Frequency (n)	Percent (%)
**Age (years)**		
< 24	214	48.1
25–34	187	42.0
> 35	44	9.9
**Marital status**		
Married	437	98.2
Others (widowed, divorced, separated & never married)	8	1.8
**Religion**		
Orthodox	185	41.6
Muslim	223	50.1
Other (Protestant)	37	8.3
**Ethnic group**		
Oromo	367	82.5
Amhara	67	15.1
Others (Gurage, Silte and Hadiya)	11	2.5
**Head of household**		
Husband	223	50.1
Wife	15	3.4
Both	207	46.5
**Maternal occupation**		
Wife	260	58.4
Farmer	12	2.7
Daily labor	23	5.2
Government employee	83	18.7
Private employee	54	12.1
Petty trade	13	2.9
**Paternal occupation**		
Farmer	58	13
Government/private employee	148	33.3
Petty trade	93	20.9
Other	146	32.8
**Maternal education**		
Illiterate	35	7.9
Some education	410	92.1
**Paternal education**		
Illiterate	24	5.4
Literate	421	94.6
**Household wealth index**		
High	137	30.8
Medium	201	45.2
Low	107	24
**Family size**		
1–3	275	61.8
4–6	144	32.4
≥ 7	26	5.8

### Utilization of iron supplements during pregnancy

**Figure 1:**
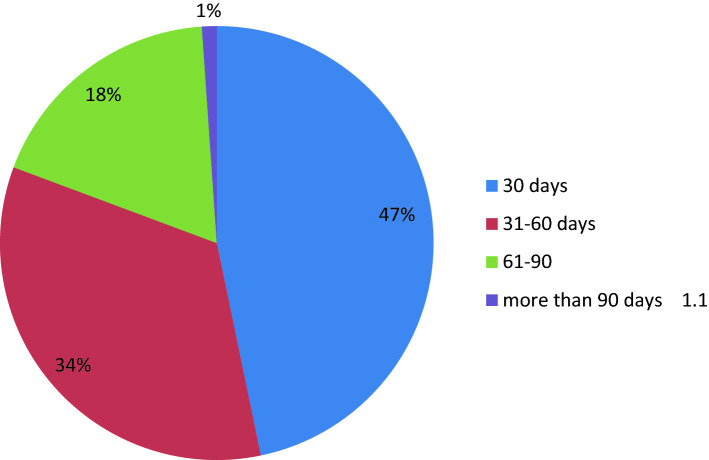
Utilization of iron supplementation among pregnant women at Robe town, Bale zone, Southeast Ethiopia, 2015 (n = 445).

Out of 445 pregnant women who were enrolled in the study, 54%; 95% CI (49.4, 58.4%), 45.2%; 95% CI (40.9, 49.4%), 4.3%; 95% CI (2.5, 6.3%), and 2.2%; 95% CI (1.1, 3.6%) of women received iron supplements during their first, second, third, and fourth antenatal care visits, respectively. Of 445 pregnant women who were given iron supplements, 46.7%; 95% CI (42.2, 51.5%), 33.9%; 95% CI (29, 37.8%), 18.2%; 95% CI (14.4, 22.2%), and 1.1%; 95% CI (0.2, 2.2%) of them received iron supplements for 30 days, 31–60 days, 61–90 days, and more than 90 days, respectively (Fig. 1).

### Compliance with iron supplements during pregnancy

The rate of compliance with iron supplementation during pregnancy was found to be 92.4%; 95% CI (89.9, 94.6%).

### Predictors of compliance with iron supplements during pregnancy

In multivariable logistic regression analysis, women’s literacy (AOR = 4.45, 95% CI 1.41, 13.99), the medium (AOR = 0.33, 95% CI 0.11, 0.98), and high (AOR = 0.18, 95% CI 0.04, 0.68) wealth quintiles, absence of discomfort or adverse effects related to iron supplements (AOR = 2.94, 95% CI 1.17, 7.39), women’s having comprehensive knowledge of anemia (AOR = 2.62, 95% CI 1.02, 6.70) and having good knowledge of iron supplements (AOR = 3.30, 95% CI 1.12, 9.76), having information about the importance of iron supplementation (AOR = 2.86, 95% CI 1.04, 7.87), being visited by urban health extension workers (AOR = 0.31, 95% CI 0.12, 0.83) were found to be significant predictors of compliance with iron supplements during pregnancy (Table 2).

**Table 2:** Predictors of compliance with iron supplementation among pregnant women at Robe town, Bale Zone, Southeast Ethiopia, 2015 (n = 445).

Variables	Compliance with iron supplementation					
	Yes	No	COR	95% CI	AOR	95%CI
	n (%)	n (%)				
**Respondent education**						
Illiterate	27 (77.1)	8 (22.9)	1		1	
literate	384 (93.7)	26 (6.3)	0.23	0.09–0.56	4.45	1.41–13.99*
**Wealth index**						
High	130 (94.9)	7 (5.1)	0.67	0.23–1.90	0.18	0.05–0.68*
Medium	182 (90.5)	19 (9.5)	0.52	0.21–1.26	0.33	0.11–0.98*
Low	99 (92.5)	8 (7.5)	1		1	
**Experienced discomfort related to iron supplement**						
Yes	146 (86.9)	22 (13.1)	1		1	
No	265 (95.7)	12 (4.3)	3.33	1.60–6.92	2.94	1.17–7.39*
**Satisfied with dose**						
Yes	346 (94.5)	20 (5.5)	3.73	1.79–7.75	1.99	0.76–5.23
No	65 (82.3)	14 (17.7)	1		1	
**Comprehensive knowledge of anemia**						
Yes	317 (95.8)	14 (4.2)	4.82	2.34–9.91	2.62	1.02–6.70*
No	94 (82.5)	20 (17.5)	1		1	
**Forgotten iron supplement intake in between**						
Yes	143 (89.9)	16 (10.1)	1		1	
No	268 (93.7)	18 (6.3)	1.67	0.82–3.37	0.94	0.38–2.36
**Thought too many iron supplement intake may harm the fetus**						
Yes	73 (88)	10 (12)	1		1	
No	338 (93.4)	24 (6.6)	0.52	0.24–1.13	1.56	0.61–3.97
**Ever interrupted iron supplement intake**						
Yes	121 (87.7)	17 (12.3)	1		1	
No	290 (94.5)	17 (5.5)	2.40	1.18–4.85	1.84	0.69–4.90
**Knowledge of iron supplementation**						
≤ 2	162 (85.7)	27 (14.3)	1		1	
≥ 3	249 (97.3)	7 (2.7)	5.93	2.53–13.93	3.30	1.12–9.76*
**Information about the importance of iron supplementation**						
Yes	335 (94.9)	18 (5.1)	3.92	1.91–8.03	2.86	1.04–7.87*
No	76 (82.6)	16 (17.4)	1		1	
**Source of iron supplement**						
Private pharmacy	47 (82.5)	10 (17.5)	1		1	
Free hospital/H/C	364 (93.8)	24 (6.2)	3.23	1.45–7.17	3.77	1.33–10.69*
**Ever been visited by UHEW**						
Yes	132 (89.2)	16 (10.8)	0.53	0.26–1.08	0.31	0.12–0.83*
No	279 (93.9)	18 (6.1)	1		1	
**Experienced serious pregnancy complications**						
Yes	59 (86.8)	9 (13.2)	1		1	
No	352 (93.4)	25 (6.6)	2.95	0.96–4.83	0.65	0.22 to1.94
**Gestational age**						
≤ 12	3 (75)	1 (25)	1		1	
13–24	92 (92)	8 (8)	3.83	0.36–41.24	0.89	0.04–20.48
≥ 25	316 (92.7)	25 (7.3)	4.20	0.42–42.00	1.11	0.06–22.60

* Statistical significant association; *COR* Crude odds ratio, *AOR* Adjusted odds ratio, *CI* Confidence interval, *1* Reference category, *UHCW* Urban healthcare worker.

## Discussion

The objective of this study was to assess utilization and compliance with iron supplementation and predictors among pregnant women. The overall utilization of iron supplementation during pregnancy was low, with a relatively high level of compliance with the supplements. Maternal formal education, knowing anemia and the importance of iron supplementation during pregnancy, not experiencing side effects related to iron tablets, and getting iron for free have positively influenced compliance with iron supplementation during pregnancy. On the other hand, having high/medium wealth quintiles and being visited by urban health extension workers were negatively associated with compliance with iron supplementation during pregnancy.

Accordingly, of pregnant women who were given iron supplements, 46.7%, 33.9%, 18.2%, and 1.1% received iron supplements for 30 days, 31–60 days, 61–90 days, and more than 90 days, respectively. This study’s finding was argued with a study conducted in Ethiopia^20^. However, fewer than one in twenty pregnant women (3.5%) took the supplements for more than 90 days. Besides, the utilization of iron supplementation was disappointingly low, as only 17.3% of women took the supplements during their most recent pregnancy in the preceding 5 years, and only 0.4% were supplemented for 90 or more days^14^. This could be due to the difference in the educational status of respondents and access to iron supplements.

In this study, the overall compliance rate of iron supplementation during pregnancy was found to be 92.4%. The current finding was higher than many previous reports in Ethiopia, such as the compliance rate, which varied from 60 to 63.6% in Addis Ababa^32,44,45^, 87.6% in Ethiopia^37^, Dire Dawa^46^, Shalla District^47^, 43.4% Dilla town^48^, 38.3% Hadassah^28^, 51.4% Burin district^49^, 52.9% Debbie^50^, 28.1% Denbiya District^36^, 28.7% Lay Armachiho^31^, 55% Gondar^51^, 47.6% Aykal town^30^, 44% Debre Tabor^52^, 43% Wollo^53^ and 74.9% in four regions of Ethiopia^20^, 67.6% Simada district^54^, 52.8% Debay Tilat Gen district^55^, 37.2% Northwestern zone of Ethiopia^35^, 55.5% Debre Markos town^56^, 76.9% Dangila, Northern Ethiopia^57^, 40.9% in Adwa town^58^, and meta-analyses, 46.15, 46.1, 41.38 and 43.63%^59–62^. The present finding was also higher than many others, for example, a meta-analysis in SSA^63^, Uganda^64^, Kenya^65^, Nigeria^24^, Northwest Tanzania^66^, Northern Tanzania^67^, 22 sub-Saharan African countries^23^, Niger^68^, West Iran^69^, Cambodia^34^, Nepal^70,71^, India^72^, and Sri Lanka^73^. Likewise, the current finding was higher than the study finding conducted in a high-income country (HIC) in which the compliance rate was 85% in Sweden^74^. However, the current finding was lower than the report from South Africa^26^ in which the compliance rate was 93%. This might be because of variation in the educational status of respondents and self-reporting as education increases pregnant women’s ability to easily access information dissemination and media outlets.

Educated pregnant women were 4.45 times more compliant with iron supplementation during pregnancy as compared to illiterate pregnant women. The current finding was agreed with studies conducted in Addis Ababa^44^, Denbiya District^36^, Lay Armachiho^31^, Debre Markos Town^56^, a meta-analysis in Ethiopia^61^. This might be because educated pregnant women were more likely to appreciate the benefits of iron supplementation in pregnancy and hence more likely to comply with the prescription. Nonetheless, the present study’s results were claimed by a study conducted in Ethiopia^20^. Similarly, the current study was supported by a study conducted in Burji Districts^49^. The possible explanation could be for a variety of reasons, despite the pregnant women's living in different settings. Furthermore, the findings of this study agreed with those of 22 subSaharan African^23^, Nigeria^24^, West Iran^69^, Nepal^70^, and Urban Slum^25^. This could be because educated pregnant women understand the benefits of iron supplementation as well as the risks that occur in the absence of iron supplementation and are therefore more compliant with iron supplementation in pregnancy. Nevertheless, the current study claims to be a study carried out in India^72^.

Pregnant women who had high wealth quintiles were 82% less likely to be compliant with iron supplementation as compared to pregnant women who had low wealth quintiles. The present study's results were agreed with those of a study conducted in Ethiopia^20^. The current study's findings are supported by studies conducted in the Northwest, Ethiopia^31^, and Nepal^70^. This could probably be because pregnant women who were socio-economically empowered thought that iron supplement intake affected them or their fetuses. However, this study disagreed with a study carried out in 22 SSA countries^23^. The possible explanation might be that pregnant women who had high wealth quantiles received information from a media outlet and understood the benefit of iron.

Pregnant women who got free iron supplements from government hospitals or health centers were 3.77 times more likely to be compliant with iron supplementation as compared to those who bought iron supplements from a private pharmacy. This current study’s results agreed with those of a study conducted in Senegal^75^. This could be because of socioeconomic status.

Pregnant women who had not experienced discomfort related to iron supplement intake were 2.94 times more compliant with iron supplementation during pregnancy as compared to pregnant women who had experienced discomfort related to iron supplement intake. The present finding agrees with a study carried out in Nigeria^24^. The possible explanation might be that educated pregnant women understand the benefits of iron supplementation and the consequences of anemia during pregnancy.

Nevertheless, this current study result was argued with a study conducted in Bangladesh^76^, Iran^77^, and Thailand^78^ which indicated that gastrointestinal side effects were not significantly associated with compliance. This discrepancy could be because of cultural beliefs.

Pregnant women who had comprehensive knowledge of anemia were 2.62 times more likely to be compliant with iron supplement intake as compared to pregnant women who didn't have comprehensive knowledge of anemia. The present study supported a study conducted in four regions of Ethiopia^20^. Likewise, the current study findings agreed with several studies carried out in Northwest Ethiopia^50^, Aykal Town^30^, North Wollo Zone^53^, and Ethiopia^60,61^. The probable reason could be the awareness and educational status of the respondents.

Pregnant women who got free iron supplements from government hospitals or health centers were 3.77 times more likely to be compliant with iron supplementation as compared to those who bought iron supplements from a private pharmacy. This current study’s results agreed with those of a study conducted in Senegal^75^. This could be because of socioeconomic status.

Pregnant women who had not experienced discomfort related to iron supplement intake were 2.94 times more compliant with iron supplementation during pregnancy as compared to pregnant women who had experienced discomfort related to iron supplement intake. The present finding agrees with a study carried out in Nigeria^24^. The possible explanation might be that educated pregnant women understand the benefits of iron supplementation and the consequences of anemia during pregnancy.

Nevertheless, this current study result was argued with a study conducted in Bangladesh^76^, Iran^77^, and Thailand^78^ which indicated that gastrointestinal side effects were not significantly associated with compliance. This discrepancy could be because of cultural beliefs.

Pregnant women who had comprehensive knowledge of anemia were 2.62 times more likely to be compliant with iron supplement intake as compared to pregnant women who didn't have comprehensive knowledge of anemia. The present study supported a study conducted in 4 regions of Ethiopia^20^. Likewise, the current study agreed with several studies carried out in Northwest Ethiopia^50^, Aykal town^30^, North Wollo Zone^53^, and Ethiopia^60,61^. The probable reasons could be because of the awareness and educational status of the respondents. The current study’s findings were also supported by studies in SSA^63^, Northwest Tanzania^66^, and Cambodia^34^. The possible explanation could be that knowledge helps a woman have a good perception of the advantages of taking an iron supplement.

Pregnant women who had good knowledge of iron supplementation were 3.30 times more likely to be compliant with iron supplements as compared to pregnant women who had poor knowledge of iron supplementation. This current study was agreed with several studies carried out in Burji District^49^, West Dembia, Ethiopia^50^, Aykal town^30^, North Wollo zone^53^, Debre Tabor^52^, Debre Markos town^56^, Ethiopia^60,61^. Likewise, the current study supported studies conducted in SSA^63^, Kenya^65^, Nigeria^24^, Muntinlupa, and the Philippines^79^. Pregnant women who had good knowledge of iron supplementation might be more likely to receive information about iron supplements and understand education messages delivered via different media outlets.

Pregnant women who had information about the importance of iron supplementation during pregnancy were 2.86 times more compliant with iron supplementation as compared to pregnant women who had not gotten information about the importance of iron supplementation during pregnancy. The current study agreed with the studies conducted in Akaki Kality^32^, Hawassa^28^, Debre Tabor^52^, Aykal Town^30^, North Wollo zone^53^, meta-analyses in Ethiopia^59–61^, a study of four regions of Ethiopia^20^, and Adwa Town^58^. The possible explanation might be that pregnant women who received information about iron supplements have increased their knowledge, attitude, and behavior.

A study conducted in SSA^63^, Uganda^22^, North West Tanzania^66^, and Iran^77^ found that most women received information on anemia and iron supplementation from health workers rather than other information sources such as the media, but their knowledge was still low regardless of the training.

### Strength and limitations

This study used a community-based cross-sectional study, and it was representative of the source population. On the other hand, recall bias might occur due to self-reporting because the gold standard (like biochemical and stool tests) had not been used, and this might have underestimated or overestimated the utilization and compliance with iron supplementation during pregnancy. The findings of this study might not be representative of the country. Since this study used a cross-sectional study, it could not establish a cause-effect relationship.

## Conclusion

This study demonstrated that the utilisation of iron supplementation during pregnancy was low, with a high level of compliance with the supplements. It was also revealed that the most common types of discomfort experienced by pregnant women who took iron supplements were gastric burning and headaches. The study also identified that being literate women, being free of charge iron supplements, not experiencing discomfort related to iron supplement intake, having comprehensive knowledge of anemia, having good knowledge of iron supplementation, and information about the importance of iron supplementation during pregnancy were factors significantly affecting compliance with iron supplementation during pregnancy. However, high wealth quintiles, medium wealth quintiles, and ever being visited by urban health extension workers were less likely to be associated with compliance with iron supplementation during pregnancy. The proper delivery of training to pregnant women on anemia causes, symptoms, consequences, and prevention, as well as the benefits of iron supplementation, is crucial. There should be a continuous and timely provision of iron supplements for health facilities. The findings of this study have implications for clinicians and policymakers. Further research with a strong study design using golden standard methods is warranted.

## Data Availability

All the relevant information is within this manuscript.
